# Decreased Expression of Placental Proteins in Recurrent Pregnancy Loss: Functional Relevance and Diagnostic Value

**DOI:** 10.3390/ijms25031865

**Published:** 2024-02-03

**Authors:** Eszter Tóth, Dániel Györffy, Máté Posta, Petronella Hupuczi, Andrea Balogh, Gábor Szalai, Gergő Orosz, László Orosz, András Szilágyi, Orsolya Oravecz, Lajos Veress, Sándor Nagy, Olga Török, Padma Murthi, Offer Erez, Zoltán Papp, Nándor Ács, Nándor Gábor Than

**Affiliations:** 1 Systems Biology of Reproduction Research Group, Institute of Molecular Life Sciences, HUN-REN Research Centre for Natural Sciences, Magyar Tudósok Körútja 2, H-1117 Budapest, Hungary; 2 Faculty of Information Technology and Bionics, Pázmány Péter Catholic University, H-1083 Budapest, Hungary; 3 Doctoral School, Semmelweis University, H-1085 Budapest, Hungary; 4 Maternity Private Clinic of Obstetrics and Gynecology, H-1126 Budapest, Hungary; 5 Department of Surgery, Medical School, University of Pécs, H-7624 Pécs, Hungary; 6 Department of Obstetrics and Gynecology, Medical School, University of Debrecen, H-4032 Debrecen, Hungary; 7 Doctoral School of Biology, ELTE Eötvös Loránd University, H-1117 Budapest, Hungary; 8 Department of Pharmacology and Pharmacotherapy, Medical School, University of Debrecen, H-4032 Debrecen, Hungary; 9 Faculty of Health and Sport Sciences, Széchenyi István University, H-9026 Győr, Hungary; 10 Department of Pharmacology, Monash Biomedicine Discovery Institute, Clayton 3168, Australia; 11 Department of Obstetrics and Gynaecology, University of Melbourne, Royal Women’s Hospital, Parkville 3052, Australia; 12 Department of Obstetrics and Gynecology, Soroka University Medical Center, Ben Gurion University of the Negev, Be’er Sheva 8410501, Israel; 13 Department of Obstetrics and Gynecology, Medical School, Wayne State University, Detroit, MI 48201, USA; 14 Department of Obstetrics and Gynecology, Medical School, Semmelweis University, 27 Baross Street, H-1088 Budapest, Hungary

**Keywords:** bioinformatics, habitual abortion, liquid biopsy, non-invasive monitoring, placental protein, prenatal diagnostics, recurrent miscarriage, spontaneous abortion

## Abstract

Miscarriages affect 50–70% of all conceptions and 15–20% of clinically recognized pregnancies. Recurrent pregnancy loss (RPL, ≥2 miscarriages) affects 1–5% of recognized pregnancies. Nevertheless, our knowledge about the etiologies and pathophysiology of RPL is incomplete, and thus, reliable diagnostic/preventive tools are not yet available. Here, we aimed to define the diagnostic value of three placental proteins for RPL: human chorionic gonadotropin free beta-subunit (free-β-hCG), pregnancy-associated plasma protein-A (PAPP-A), and placental growth factor (PlGF). Blood samples were collected from women with RPL (*n* = 14) and controls undergoing elective termination of pregnancy (*n* = 30) at the time of surgery. Maternal serum protein concentrations were measured by BRAHMS KRYPTOR Analyzer. Daily multiple of median (dMoM) values were calculated for gestational age-specific normalization. To obtain classifiers, logistic regression analysis was performed, and ROC curves were calculated. There were differences in changes of maternal serum protein concentrations with advancing healthy gestation. Between 6 and 13 weeks, women with RPL had lower concentrations and dMoMs of free β-hCG, PAPP-A, and PlGF than controls. PAPP-A dMoM had the best discriminative properties (AUC = 0.880). Between 9 and 13 weeks, discriminative properties of all protein dMoMs were excellent (free β-hCG: AUC = 0.975; PAPP-A: AUC = 0.998; PlGF: AUC = 0.924). In conclusion, free-β-hCG and PAPP-A are valuable biomarkers for RPL, especially between 9 and 13 weeks. Their decreased concentrations indicate the deterioration of placental functions, while lower PlGF levels indicate problems with placental angiogenesis after 9 weeks.

## 1. Introduction

Miscarriage is defined as the loss of pregnancy before the 20^th^ week of gestation [1,2], affecting 50–70% of all gestations and 15–20% of clinically recognized pregnancies [3,4,5,6,7,8,9,10,11,12,13,14,15]. Moreover, the risk of miscarriage is directly related to the number of previous miscarriages [14,15,16]. As a consequence, recurrent pregnancy loss (RPL), which is defined by the WHO as the loss of three or more consecutive pregnancies before 20th weeks of gestation [6,10,17,18,19], affects 1–5% of pregnancies [6,10,19,20,21]. As the risk of miscarriage in a subsequent pregnancy is 30% after two pregnancy losses and 33% after three losses [21], the American Society for Reproductive Medicine redefined RPL as two or more failed clinical pregnancies [19,22]. Altogether, RPL has critical importance and enormous demographical, social, psychological, and economic impact [23,24], especially in most developed countries, where a continuous decline in reproductive rates has been observed since the 1960s.

Additional risk factors of RPL include maternal age [10]; genetic- [6,17,25,26,27,28,29,30], endocrine- [6,17,31,32,33,34,35], anatomic- [6,28,36,37,38], immunologic- [6,39,40,41,42,43,44,45], and hemostatic disorders [6,46,47,48,49]; as well as antiphospholipid syndrome [6,17,50,51,52]. However, about half of the cases of RPL have no evident causes and molecular background [6,13,19,22,53]. In light of the syndromic nature of RPL, along with the lack of comprehensive molecular pathophysiology, early and reliable prediction and prevention of RPL are still some of the largest challenges in reproductive medicine.

Currently, the detection of early pregnancy failures includes an ultrasound scan and the determination of maternal blood concentrations of different biomarkers measured either alone or in combinations. However, there is still no unified protocol or agreement on the prediction of RPL. The diagnostic or predictive value of biomarkers related to the underlying primary clinical disease leading to RPL, like immunological, thrombophilia, or endocrine markers [4,53], are limited, and they are not specific to RPL. Protein biomarker studies for RPL have been performed either on non-pregnant women for risk assessment [34,54,55,56,57,58,59,60] or on pregnant women to predict the outcome of the current pregnancy [43,61,62,63,64,65,66,67,68,69,70,71,72,73,74]. However, the results of these studies are conflicting and most often not comparable, mainly because of heterogeneous or inadequate definitions and patient groups, as well as differences in methodologies [75,76].

Altogether, a reliable method to predict RPL with high confidence in the early stage of pregnancy to enable preventive therapies remains elusive. Therefore, investigations on known as well as new and more effective biomarkers are warranted in well-designed studies that apply strict clinical definitions, homogeneous patient groups, and good-quality samples. Standardized sample collection and sample treatment, as well as data evaluation, are also very important to identify better biomarker candidates and to define the exact classifier and predictive values of biomarker proteins. Since placental functions are severely disturbed in miscarriages [77,78,79,80,81,82,83], here, our aim was to determine the changes in concentrations of three known placental biomarker proteins: the free beta-subunit of human chorionic gonadotropin (free β-hCG), pregnancy-associated plasma protein-A (PAPP-A), and placental growth factor (PlGF), and their combinations in women with RPL. Our study utilized samples collected under strict biobanking protocols from homogenous patient groups; immunoassays were performed according to international clinical standards, data were normalized to large population standards, and reliable analytical and bioinformatics methods were used.

## 2. Results

### 2.1. The Expression Patterns of hCG, PlGF, and PAPPA Proteins

**Figure 1:**
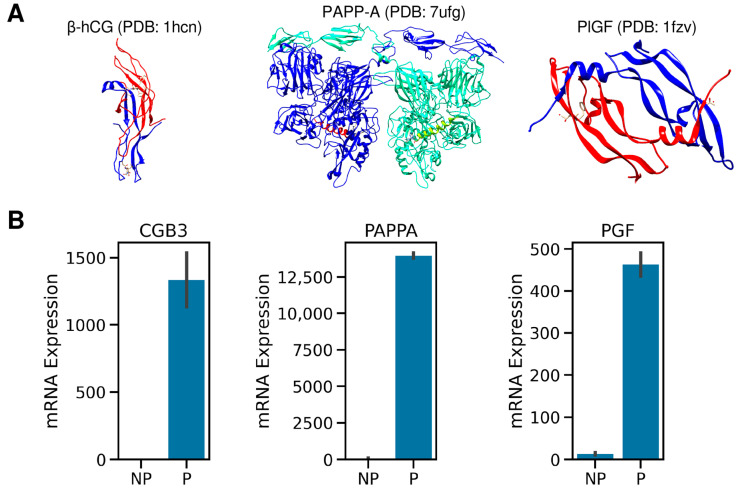
(**A**) The three-dimensional structures of β-hCG (PDB: 1hcn), PAPP-A (PDB: 7ufg), and PlGF (PDB: 1fzv) from the Protein Data Bank. (**B**) Comparison of the mRNA expression levels of β-HCG (*CGB3* gene), PAPP-A (*PAPPA* gene), and PlGF (*PGF* gene) in the placenta vs. 83 non-placental tissues from the GeneAtlas U133A data set. The error bars represent the 95% confidence interval of the mean. Human chorionic gonadotropin free beta-subunit, free β-hCG; placental growth factor, PlGF; pregnancy-associated plasma protein A, PAPP-A; placental tissue, P; mean of 83 non-placental tissue, NP.

The genes encoding the hCG, PAPP-A, and PlGF proteins are predominantly expressed in the placenta, based on mRNA expression data for 84 tissue types in the GeneAtlas U133A data set [84,85,86] (Figure 1 and Supplementary Figure S1). The mRNA expression levels of the *CGB3*, *PGF*, and *PAPPA* in placental tissue are 252×, 47×, and 1746× fold larger than the medians of their expression in 83 other tissue types, respectively. The Pearson correlation coefficients between the tissue-wise expression levels of the three genes in all combinations are >0.9.

### 2.2. Demographic and Clinical Data

Demographic and clinical characteristics of the study groups are displayed in Table 1. Maternal age and gravidity were higher in RPL than in controls. Women had from one to three previous pregnancy losses in the RPL group.

### 2.3. Gestational Age-Specific Distribution of Data

**Figure 2:**
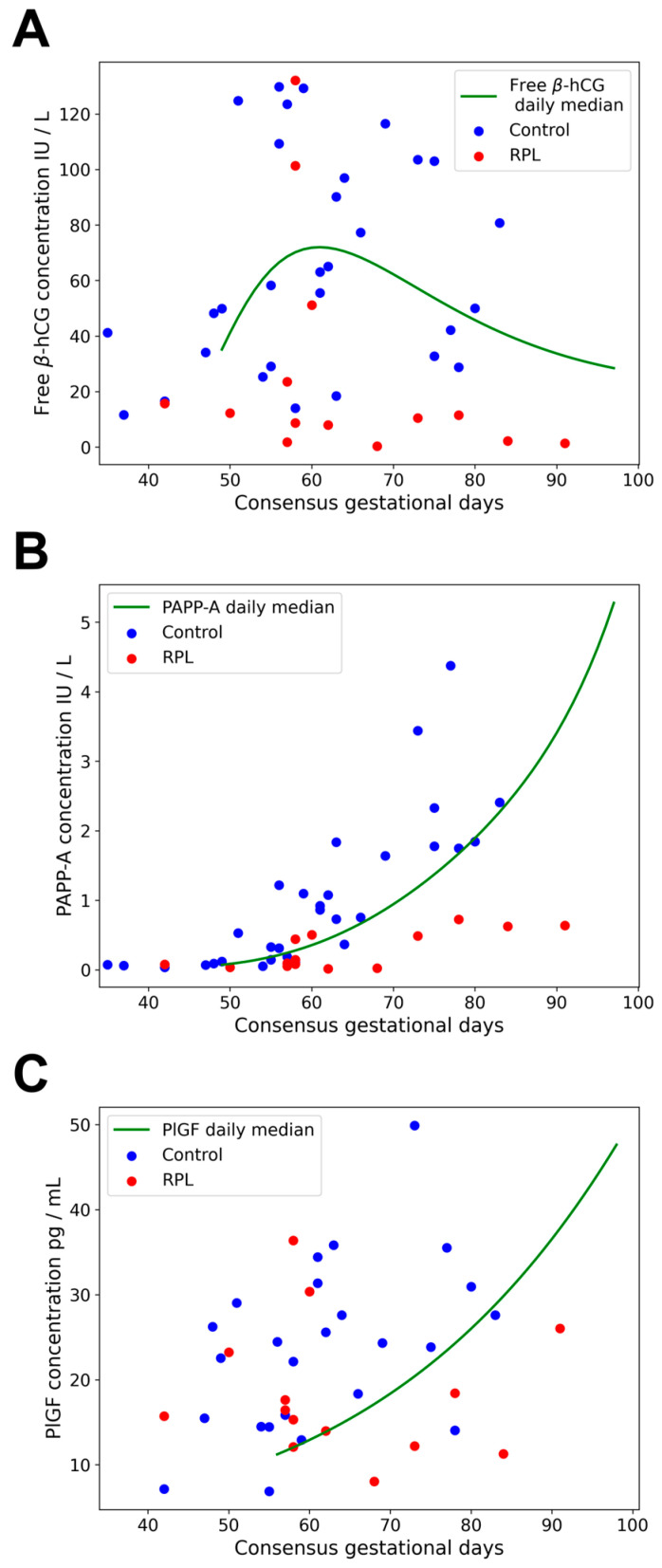
Maternal serum concentrations of free β-hCG, PAPP-A, and PlGF proteins compared to daily median curves. Maternal serum concentrations of free β-hCG (**A**), PAPP-A (**B**), and PlGF (**C**) proteins in the RPL group (*n* = 14) and the control group (*n* = 30) were plotted against consensus gestational days. Daily median reference concentrations (green), used for gestational age-specific normalization, were calculated based on Wright et al. [87] for free β-hCG and PAPP-A (*n* = 222,475), and based on Tsiakkas et al. [88] for PlGF (*n* = 38,002). The reference values were obtained from Thermo Fisher Scientific. Human chorionic gonadotropin free beta-subunit, free β-hCG; placental growth factor, PlGF; pregnancy-associated plasma protein A, PAPP-A; recurrent pregnancy loss, RPL.

Samples were collected between 42 and 91 gestational days in the RPL group and between 35 and 83 gestational days in the control group. Inside of the specified ranges, the distribution of data points is depicted in Figure 2. The daily median reference values of free β-hCG and PAPP-A were available for 49–97 gestational days based on 222,475 patients, and of PlGF, for 56–98 days based on 38,002 patients [87,88]. These daily medians were applied as reference values during gestational age-specific normalization when calculating dMoM values.

Gestational age-specific normalization was performed because the concentration of each protein varies with gestational age [89]. Daily medians for PAPP-A and PlGF concentrations monotonically increased in the gestational age range for which daily median data were available, while the concentration of free β-hCG first increased and then decreased in the investigated gestational age range, having a maximum value at the 61^st^ day of gestation. The previously published equations [87,88] describing the log_10_ daily median values for PAPP-A, free β-hCG, and PlGF concentrations as a function of gestational age are:

$$\log_{10}\mathrm{PAPP}-A=0.1950+2.844\times 10^{-2}\times \left(GA-77\right)-3.522\times 10^{-4}\times {\left(GA-77\right)}^{2}+1.244\times 10^{-5}\times {\left(GA-77\right)}^{3},$$

$$\log_{10}\mathrm{free}\;\beta -\mathrm{hCG}=-3.240-5.097\times 10^{-2}\times \left(GA-77\right)-4.480\times 10^{-4}\times {\left(GA-77\right)}^{2}+3.152\times \log_{10}\left(GA-40\right)$$

and

$$\log_{10}\mathrm{PlGF}=1.319+0.01506\times \left(GA-77\right)-1.363\times 10^{-5}{\times \left(GA-77\right)}^{2}-2.336\times 10^{-7}{\times \left(GA-77\right)}^{3}$$

Reference values (daily median curves) are shown as green lines in Figure 2 and are listed in Supplementary Table S1, respectively.

### 2.4. Maternal Serum Concentrations of Free β-hCG, PAPP-A, and PlGF in RPL and controls

**Figure 3:**
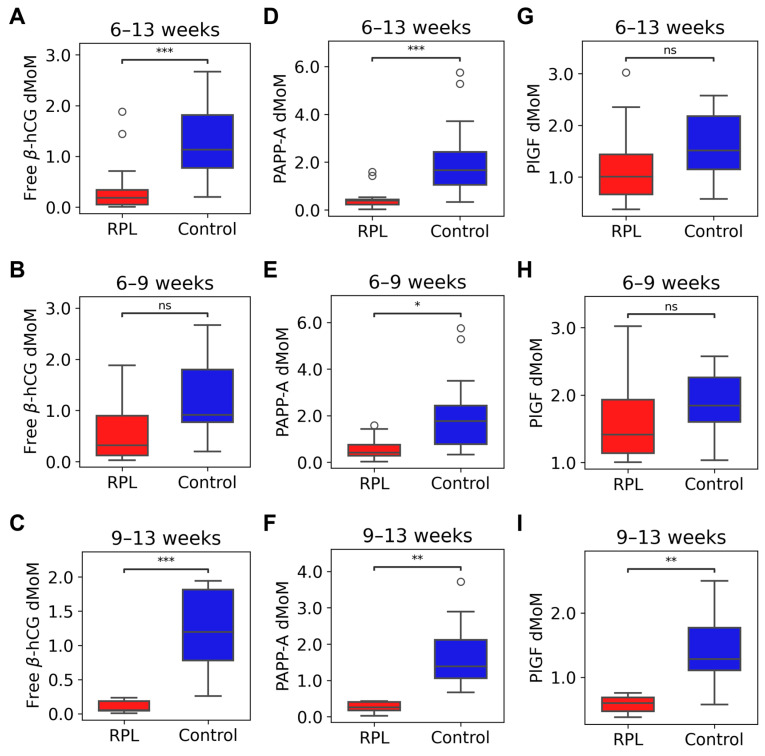
Box plots for free β-hCG, PAPP-A, and PlGF dMoMs in the study groups. Box plots represent dMoM values of free β-hCG protein in the RPL (*n* = 13) and control (*n* = 25) groups in the whole gestational range (**A**), between 6 and 9 weeks of gestation (RPL: *n* = 8, control: *n* = 13) (**B**), and between 9 and 13 weeks of gestation (RPL: *n* = 5, control: *n* = 12) (**C**), PAPP-A protein in the RPL (*n* = 13) and control (*n* = 25) groups in the whole gestational range (**D**), between 6 and 9 weeks of gestation (RPL: *n* = 8, control: *n* = 13) (**E**), and between 9 and 13 weeks of gestation (RPL: *n* = 5, control: *n* = 12) (**F**), PlGF protein in the RPL (*n* = 12) and control (*n* = 20) groups in the whole gestational range (**G**), between 6 and 9 weeks of gestation (RPL: *n* = 7, control: *n* = 8) (**H**), and between 9 and 13 weeks of gestation (RPL: *n* = 5, control: *n* = 12) (**I**). Significance levels denoted as follows: ns: *p* > 0.05, *: *p* < 0.05, **: *p* < 0.01, ***: *p* < 0.001. Daily multiple of median, dMoM; human chorionic gonadotropin free beta-subunit, free β-hCG; placental growth factor, PlGF; pregnancy-associated plasma protein A, PAPP-A; recurrent pregnancy loss, RPL.

The mean maternal serum concentration of all proteins was lower in RPL compared to controls (free β-hCG RPL: 10.96 IU/L, control: 56.91 IU/L, *p* = 2.91 × 10^−4^; PAPP-A RPL: 0.12 IU/L, control: 0.74 IU/L, *p* = 5.27 × 10^−3^; PlGF RPL: 16.07 pg/mL, control: 24.32 pg/mL, *p* = 0.0106). After gestational age-specific normalization, PAPP-A and PlGF but not free β-hCG dMoMs had more significant differences between the groups than concentrations (free β-hCG RPL: 0.18, control: 1.13, *p* = 4.61 × 10^−4^, Figure 3A; PAPP-A RPL: 0.39, control: 1.66, *p* = 9.13 × 10^−5^, Figure 3D; PlGF RPL: 1.01, control: 1.52, *p* = 5.27 × 10^−3^, Figure 3G).

Since the concentrations of these proteins change during normal pregnancy, we hypothesized that these gestational age-dependent changes also occur in RPL. Therefore, we analyzed the data in two gestational age ranges, between 6 and 9 and between 9 and 13 weeks.

Between 6 and 9 weeks, the mean maternal serum concentration of free β-hCG (RPL: 15.70 IU/L, control: 52.75 IU/L, *p* = 0.0380) and dMoM (RPL: 0.32, control: 0.91, *p* = 0.0186, Figure 3B) were lower in RPL compared to controls. The mean maternal serum concentration of PAPP-A (RPL: 0.08 IU/L, control: 0.17 IU/L, *p* = 0.0646) was not different, while PAPP-A dMoM (RPL: 0.41, control: 1.77, *p* = 5.45 × 10^−3^, Figure 3E) was lower in RPL compared to controls. The mean maternal serum concentration of PlGF (RPL: 16.42 pg/mL, control: 22.12 pg/mL, *p* = 0.244) and dMoM (RPL: 1.41, control: 1.85, *p* = 0.0584, Figure 3H) were not different in RPL compared to controls.

Between 9 and 13 weeks, the mean maternal serum concentration of all proteins was lower in RPL compared to controls (free β-hCG RPL: 2.17 IU/L, control: 79.04 IU/L, *p* = 9.37 × 10^−4^; PAPP-A RPL: 0.63 IU/L, control: 1.81 IU/L, *p* = 3.60 × 10^−3^; PlGF RPL: 12.20 pg/mL, control: 27.61 pg/mL, *p* = 6.59 × 10^−3^). Also, dMoM values of all proteins were lower in RPL compared to controls (free β-hCG RPL: 0.05, control: 1.2, *p* = 9.37 × 10^−4^, Figure 3C; PAPP-A RPL: 0.26, control: 1.39, *p* = 9.37 × 10^−4^, Figure 3F; PlGF RPL: 0.6, control: 1.28, *p* = 2.61 × 10^−3^, Figure 3I).

### 2.5. Discriminative Properties of Biomarker Proteins

Table 2, Table 3, Table 4 and Table 5 contain area under the curve (AUC), as well as sensitivities (true-positive rates), at 5% and 10% false-positive rate (FPR) values averaged over 50 runs of five-fold cross-validation, characterizing the discriminative value of proteins or their combinations. AUC, as well as sensitivities at 5% and 10% FPR values, were calculated both for serum concentrations and dMoM values.

**Figure 4:**
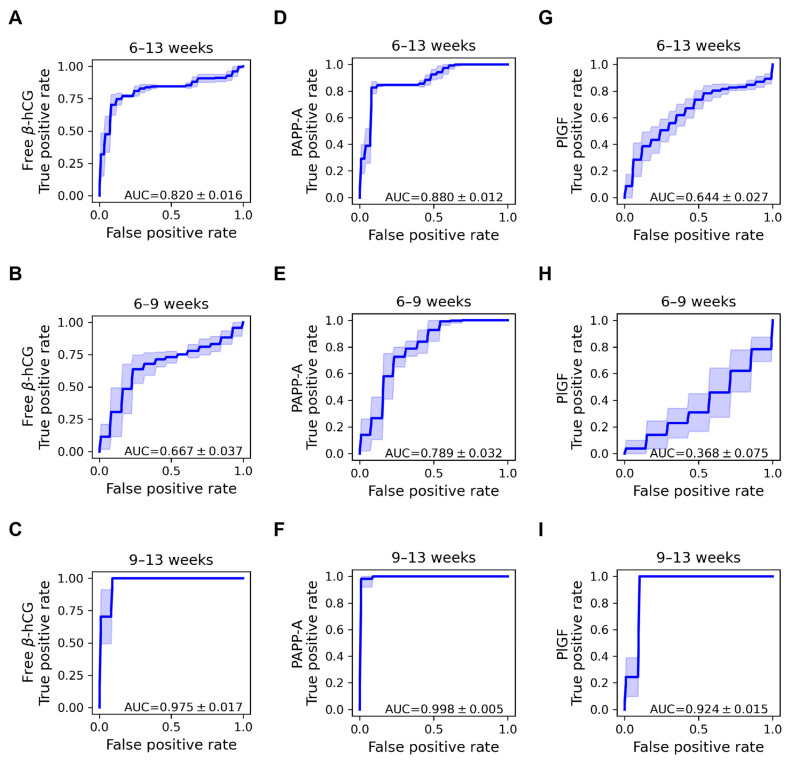
Receiver operating characteristic curves of free β-hCG, PAPP-A, and PlGF proteins in the classification of recurrent pregnancy loss. ROC curves were calculated using logistic regression analysis for log_2_ dMoM values of free β-hCG (**A**–**C**), PAPP-A (**D**–**F**), and PlGF (**G**–**I**) across the entire gestational age range (**A**,**D**,**G**), between 6 and 9 gestational weeks (**B**,**E**,**H**), and between 9 and 13 gestational weeks (**C**,**F**,**I**). The average ROC curves were obtained by averaging sensitivities at different false-positive rate values. Areas between the average TPR ± 1 standard deviation are also shown. Area under the curve, AUC; base two logarithm of daily multiple of medians, log_2_ dMoM; human chorionic gonadotropin free beta-subunit, free β-hCG; Placental growth factor, PlGF; pregnancy-associated plasma protein A, PAPP-A; receiver operating characteristic curve, ROC; true-positive rate, TPR.

**Figure 5:**
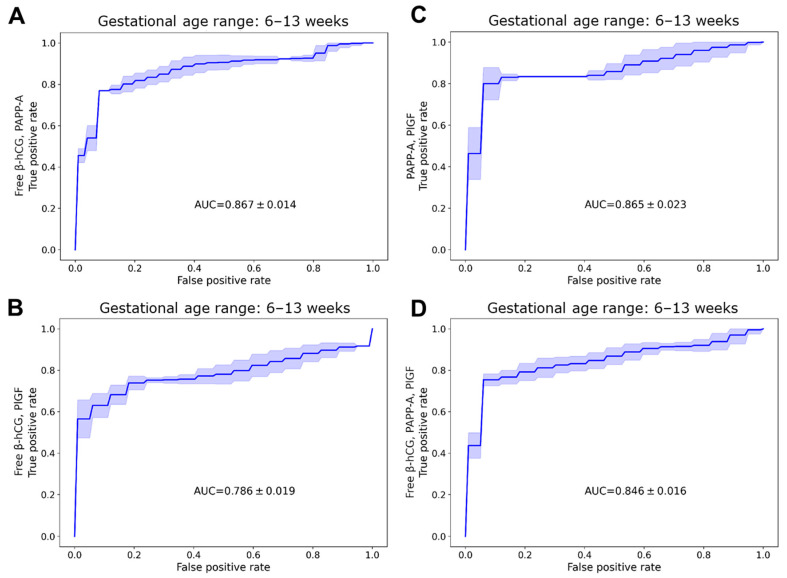
Receiver operating characteristic curves of protein combinations in the classification of recurrent pregnancy loss. ROC curves were calculated using logistic regression analysis for log_2_ dMoM values of different biomarker protein combinations in the whole gestational age range: free β-hCG and PAPP-A (**A**), free β-hCG and PlGF (**B**), PAPP-A and PlGF (**C**), as well as free β-hCG, PAPP-A, and PlGF (**D**). The average ROC curves were obtained by averaging sensitivities at different false-positive rate values. Areas between the average TPR ± 1 standard deviation are also shown. Area under the curve, AUC; base two logarithm of daily multiple of medians, log_2_ dMoM; human chorionic gonadotropin free beta-subunit, free β-hCG; placental growth factor, PlGF; pregnancy-associated plasma protein A, PAPP-A; receiver operating characteristic curve, ROC; true-positive rate, TPR.

When looking at the whole gestational age range at the single protein level (Table 2), the discriminative value of PAPP-A dMoM (AUC = 0.880, Figure 4D) was the highest, and the value of free β-hCG dMoM was relatively high as well (AUC = 0.820, Figure 4A). At the protein combination level, the discriminative values of PAPP-A dMoMs (AUC = 0.865, 0.867, 0.846, Figure 5, Table 5) were the highest. Overall, the classifier property of PAPP-A dMoM as a single protein was better than any of its combinations. In this regard, PlGF dMoM as a single protein was much less valuable (AUC = 0.644, Figure 4G), and PlGF reduced the overall discriminative value in all combinations of dMoMs (Table 5).

Between 6 and 9 weeks, the classifier properties of all proteins were poorer than for the whole gestational age range (Table 3). Briefly, PAPP-A dMoM was also the best (AUC = 0.789, Figure 4E), free β-hCG had modest discriminative values (AUC = 0.667, Figure 4B), while PlGF was a poor classifier (AUC = 0.368, Figure 4H).

Between 9 and 13 weeks, the classifier properties (Table 4) of all dMoMs were excellent (free β-hCG: AUC = 0.975, Figure 4C; PAPP-A: AUC = 0.998, Figure 4F; PlGF: AUC = 0.924, Figure 4I). In accordance, we also found that the likelihood ratios were diagnostically relevant in this interval, especially in the case of dMoMs at 10% FPR (positive likelihood ratio: 10.0, negative likelihood ratio: 0.00, respectively).

Table 6 shows all models trained on intensities or dMoM values of proteins and their various combinations.

## 3. Discussion

### 3.1. Principal Findings of the Study

(1) We corroborated earlier findings that serum concentration of free β-hCG declines after an initial increase, while the concentration of PAPP-A and PlGF monotonically increases with gestational age in the first trimester. (2) Maternal serum concentrations and gestational age-specific dMoMs of all three proteins were lower in RPL compared to controls. (3) The highest discriminative value was found for PAPP-A dMoM, both as a single analyte and in combination with other proteins within the entire gestational age range. (4) Serum concentrations and dMoMs of free β-hCG, PAPP-A, and PlGF had a larger difference between cases and controls between 9 and 13 weeks of gestation. (5) Within this period, all three proteins had excellent classifier properties for RPL.

### 3.2. Placenta-Specific Proteins

The placenta has a key role in maintaining pregnancy and supporting the developing fetus in many ways, for example, by providing nutrition, gas, and waste exchange, as well as hormonal and immunological regulation [90,91,92,93]. The failure of placental functions has a central role in the pathogenesis of many pregnancy complications such as preeclampsia [77,86,94,95,96,97,98,99,100], miscarriage [77,78,79,80,81,82,83], and RPL [83,101,102,103]. Therefore, the non-invasive monitoring of placental functions is of major importance in the early detection and prediction of these diseases. Since the early attempts of pioneers in this field in the 1960s and 1970s [104], placental functions have been evaluated by measuring placental proteins in maternal circulation. By the meticulous work of Dr. Hans Bohn and his peers, several dozens of high-abundance proteins were purified from the placenta, and antisera were raised against them, which enabled the construction of immunoassays for their measurement in circulation [104]. Of importance, due to recent technological developments, proteomics technologies have enabled the parallel investigation of thousands of proteins in the placenta and their entering into maternal blood. Indeed, the Human Protein Atlas shows that 64% (*n* = 13,003) of all human proteins (*n* = 20,162) are expressed in the placenta [105], and a lot of them are secreted into the maternal circulation as hormones, growth factors, and immune and other proteins that play a major role in the resetting of the maternal metabolic and immune homeostasis [104,106,107,108,109,110,111,112].

**Figure 6:**
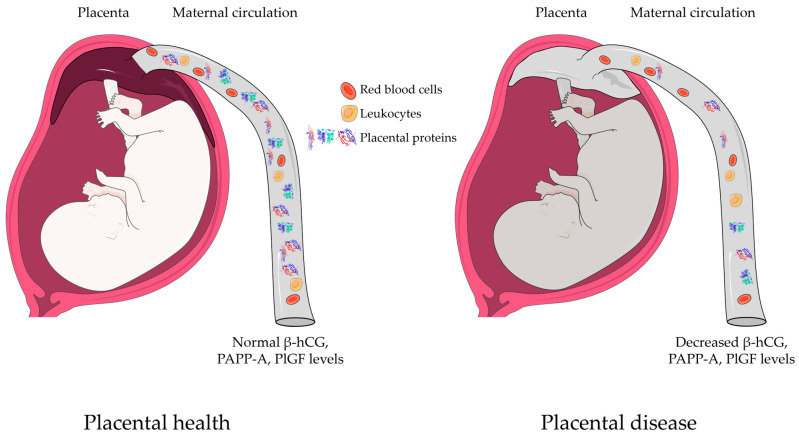
In certain placental diseases, such as recurrent pregnancy loss, there are decreased amounts of some placental proteins (e.g., β-hCG, PAPP-A, PlGF) in the maternal circulation, offering liquid-biopsy-based diagnostic potential for disease development. Human chorionic gonadotropin free beta-subunit, free β-hCG; placental growth factor, PlGF; pregnancy-associated plasma protein A, PAPP-A.

Many of these proteins are specific for the placenta, and thus, they are of paramount importance for the specific monitoring of placental functions in the maternal blood, similar to liquid biopsy of tumors [113,114,115,116]. A set of placenta-specific proteins was recently defined as proteins encoded by predominantly placenta-expressed genes by Than et al. [86] and Szilagyi et al. [85]. Our previous results confirmed that the impairment of placental functions is usually associated with the altered expression of placenta-specific proteins [104,117,118,119,120,121,122,123,124,125]. Therefore, assaying maternal blood for these placenta-specific proteins may provide information about the actual condition of the placenta in pregnancy complications (Figure 6). Of these 164 placenta-specific proteins, here, we examined free β-hCG, PAPP-A, and PlGF since these have already been used in clinical practice for the screening of preeclampsia and fetal trisomies [126,127,128,129,130,131,132,133,134,135,136,137,138].

### 3.3. Biomarker Proteins in Miscarriage and RPL

HCG is composed of two subunits (α and β) from which β-hCG is placenta-specific. HCG has a central role in the establishment and maintenance of pregnancy by many means, including the stimulation of progesterone production by the corpus luteum [139]. This is the earliest detectable marker of pregnancy [140], and the most often studied protein in the context of the prediction of miscarriage according to the systematic review and meta-analysis by Pillai et al. [141]. However, Pillai et al. also showed that β-hCG has poor sensitivity but high specificity for miscarriage [141]. Of interest, maternal serum concentrations of hCG or β-hCG were found to be reduced in RPL [61,62,63,64,65,67,68].

PAPP-A is also a placenta-specific protein, which has metalloproteinase activity and cleaves insulin-like growth factor-binding protein (IGFBP-4 and IGFBP-5), resulting in the release of bound IGF [142,143,144,145,146]. Pillai et al. reported that PAPP-A has high specificity but poor sensitivity for the prediction of miscarriage [141]. Interestingly, PAPP-A mRNA and protein expression are reduced in decidual cells in RPL [147]. However, contradicting results have been published for maternal blood, as slightly increased maternal serum PAPP-A levels were measured with ELISA in the first trimester in RPL [67], while the proteomic discovery study of maternal serum did not find PAPP-A among differentially expressed proteins in RPL [61].

PlGF is a member of the vascular endothelial growth factor family (VEGF), and by stimulating cell proliferation and migration, it plays an important role in angiogenesis as well as endothelial and tumor cell growth [142,148]. PlGF is also a predominantly placenta-expressed protein, but it is also expressed in the thyroid gland, uterine cervix, uterine, fallopian tube, and other tissues [105,149,150]. At the maternal–fetal interface, PlGF regulates decidual vascularization and angiogenesis in early human pregnancy [151], a process that is altered in different types of miscarriages [152]. However, Plaisier et al. found no significant differences in PlGF expression in the decidua in miscarriage [153]. Of note, maternal serum PlGF concentration was decreased in miscarriages or threatened abortions [154,155,156,157]; however, in the proteomic discovery study of Cui et al., PlGF was not among the differentially expressed proteins in RPL [61]. This is consistent with conflicting results of vascular endothelial growth factor expression in recurrent miscarriage [158].

### 3.4. Concentration Changes of Biomarker Proteins in RPL

Due to the failing function of the placenta in miscarriages, we expected to detect a decrease in placental protein concentrations in RPL. It was thus not surprising that we found decreased serum concentrations of free β-hCG, PAPP-A, and PlGF when assessing the 6–13 gestational week range. It is known that the concentration of individual placental proteins changes with gestational age in the maternal circulation; therefore, their serum concentration values should be compared to gestational age-matched normal values [159,160,161,162,163,164]. To achieve more accurate comparisons, here, we also performed the normalization of concentration values to population-based standard medians obtained from large patient populations. After the normalization and generation of dMoM values, we observed more significant differences between the groups for PAPP-A and PlGF dMoMs than for their concentrations. In the case of free β-hCG, the effect of normalization made the differences less, but still significant, between the groups. This is certainly due to the wide variation of individual hCG levels in the maternal serum and therefore difficulties in normalization. For example, total hCG values vary by 704-fold in the 5th week of gestation (from 1.86 to 1308 ng/mL) and by 11-fold between the 11^th^ and 13^th^ week of gestation (from 1440 to 15,318 ng/mL [165]. In addition, there are also large differences in hCG levels according to glycosylation status and various isoforms where low hyperglycosylated hCG concentrations are associated with pregnancy failure [166,167].

Since there is a rapid placental development in the first trimester which can be divided into different stages based on various parameters, including placental vascularization [168], trophoblast invasion [169], and others, we took this into account in our further analyses to achieve more accurate gestational age-specific assessments. Importantly, the establishment of placental circulation is limited by the end of the second month to protect the developing embryo and placenta from excessive oxygen exposure during organogenesis, and then placental circulation develops starting from the third month, coinciding with the establishment of the arterial inflow into the intervillous space, typically occurring between 8 and 10 weeks [168,170].

Since these changes must significantly affect the production and transport of these three placental proteins into the maternal circulation, we assessed these proteins in two gestational age sub-ranges, between 6 and 9 weeks and between 9 and 13 weeks. We found that differences in the first range were smaller, while in the second range, they were larger for all biomarker proteins. This is in accord with the lower production and transport of these proteins into the maternal circulation even in normal healthy pregnancies at 6–9 weeks of gestation when placental circulation is not yet established, which leads to smaller differences between cases and controls in this gestational age range.

Nevertheless, it is striking that dMoMs of free β-hCG and PAPP-A were lower already in early RPL cases, while the difference in PlGF dMoM was found only in late first trimester RPL cases. Therefore, the decreased levels of free β-hCG and PAPP-A in early RPL cases may indicate the deterioration of their fundamental placental functions in early RPL, while decreased PlGF level in late RPL cases may indicate that PlGF functions, including angiogenesis, are affected only in pregnancies failing after the second month when placental angiogenesis starts [168]. This phenomenon was also seen in cases of fetal death and stillbirth [171,172], possibly associated with placental bed disorders [173,174,175]. Our data suggest that the pathologic pathways in RPL include the failure of placental functions already in early RPL and the failure of angiogenesis in late RPL.

The biomarker classifier properties of these three proteins, characterized by their AUC and sensitivity (TPR) values, were closely associated with the extent of changes in their serum concentrations and dMoMs in RPL. For the entire gestational age period, the discriminative power of free β-hCG and PAPP-A, alone or in combination, was found to be much better than that of PlGF. Of interest, the best discriminatory values were found for PAPP-A, which was a novel result compared to data in the literature [61,67]. For the 6–9-week range, the classifier properties of PAPP-A were good and modest of free β-hCG, while for the 9–13-week range, all proteins had excellent biomarker properties. The clinical relevance of these investigated proteins between 9 and 13 weeks of gestation is also underscored by their positive and negative likelihood ratios, which exceeded 10 for positive test results and were below 0.2 for negative results, respectively.

### 3.5. Strengths and Limitations of the Study

The strengths of the study are: (1) strict clinical definitions and homogenous patient groups; (2) standardized sample collection protocol based on international criteria; (3) sample storage in a biobank that meets industrial standards; (4) sensitive, reliable, and robust immunoassay analysis using adjusted ELISA methodology; (5) data normalized to large population standards; and (6) reliable analytical methods.

The limitations of the study are: (1) the relatively modest number of cases in the RPL group; (2) the use of international standards for gestational age-specific mean placental protein concentrations due to the current non-availability of similar standards in Hungary, and (3) the collection of blood samples at the time of surgery when pregnancies already failed.

Since all proteins had lower serum concentrations in RPL than in controls while blood samples were collected after the embryos died in utero, the question may arise that there is a bias due to embryonic death, which may lead to lower concentrations of these analytes. However, several lines of evidence have previously shown that: (1) placentas are still viable, and placental parenchyma is unperturbed shortly after miscarriage or fetal demise due to persistent maternal perfusion [176]; (2) the placenta and trophoblasts can even persist without a fetus in molar pregnancies or choriocarcinoma, for which elevated hCG level is a good biomarker [177,178]; (3) the placental proteome contains two-times more upregulated than downregulated proteins in RPL [101]; and (4) pregnancies ending in miscarriage have smaller trophoblast volumes and reduced trophoblast growth than normal pregnancies [179].

Therefore, our results may rather point to the failed trophoblastic and placental development and functions in RPL than the effect of embryonic death, suggesting that similar changes may be seen in the levels of these biomarkers before embryonic death occurs. In this regard, it would be essential to evaluate the predictive properties of these biomarkers on blood samples collected before pregnancies failed; however, this was not possible in our current study. Therefore, future, prospective studies of RPL patients would need to investigate whether placental biomarkers also have predictive power for RPL before pregnancies fail.

### 3.6. Implications and Future Directions

There are several research and clinical implications of our study, which stem from its strengths and limitations. *Clinical implications*: While β -hCG has long been recognized as a marker for miscarriage [180], our findings suggest that PAPP-A may be better biomarker for recurrent pregnancy loss. Moreover, our study highlights that the combination of biomarkers may enhance the sensitivity and specificity of diagnostic methods over the utilization of individual biomarkers. As a broader clinical implication, our study underscores the significance of assessing placenta-specific proteins as potential diagnostic markers for RPL.

*Research implications*: Here, we investigated—in a targeted fashion—already known placental biomarker proteins which did not allow the exploration of potentially even better biomarkers or their combinations. Therefore, the incorporation of non-targeted, high-dimensional proteomics methods is encouraged for the analysis of molecular pathways of recurrent pregnancy loss and their potentially novel biomarkers. Indeed, there has been an increasing amount of data showing the involvement of immune pathways in the etiology of RPL [181,182]. In addition, larger case-control and cohort studies are needed to: (1) validate these biomarkers as diagnostic or predictive tools in recurrent pregnancy loss; (2) explore their value in different stages of pregnancy (i.e., between 6 and 9 weeks or between 9 and 13 weeks); (3) investigate the generalizability of these findings in different patient populations that various ethnic backgrounds.

## 4. Materials and Methods

### 4.1. Study Groups, Clinical Definitions, and Sample Collection

Blood samples were collected from subjects enrolled in two study groups: (1) women who had recurrent pregnancy loss (RPL, *n* = 14), and (2) as a control group, women who underwent elective termination of pregnancy at their request for non-medical reasons (*n* = 30). Samples were collected at the Maternity Private Clinic of Obstetrics and Gynecology (Budapest, Hungary) at the time of surgery.

Gestational age was determined by ultrasound scans and samples were collected within the 6–13 weeks gestational age range. Exclusion criteria for both groups included twin pregnancies or pregnancies with congenital or chromosomal abnormalities. All women in our cohort were included in the RPL group (*n* = 14) if they had two or more failed clinical pregnancies according to the definition of the American Society for Reproductive Medicine [22]. RPL cases were recruited from patients with a nonviable intrauterine pregnancy detected by ultrasound (gestational sac containing an embryo or fetus without fetal heart activity within the first 12 6/7 weeks of gestation according to the American College of Obstetricians and Gynecologists Practice Bulletin [183]). Previously failed first trimester pregnancies were complete/incomplete spontaneous or missed abortions. At least two controls were matched to each case (*n* = 30) within one week of gestation for comparability. Table 1 contains clinical and demographic information for the study groups.

Blood samples were immediately processed after sample collection. Serum was collected following blood centrifugation for 10 min at 4 °C, aliquoted, and stored at −80 °C.

### 4.2. Immunoassays

Free β-hCG, PAPP-A, and PlGF concentrations in the maternal serum were measured using a BRAHMS plus KRYPTOR Analyzer (Thermo Fisher Scientific, Waltham, MA, USA). The measurement principle was based on the TRACE™ (Time-Resolved Amplified Cryptate Emission) technology, which uses the transfer of non-radioactive energy from a donor (cage structure with a europium ion in the center (cryptate)) to an acceptor which is part of a chemically modified photo-receptive algal protein (XL665). Both cryptate and XL665 were conjugated to monoclonal antibodies targeted to different epitopes on the analytes to be measured. The proximity of the donor and acceptor, when they are part of an immunocomplex, and the spectral overlap between donor emission and acceptor absorption spectra intensify the fluorescent signal of the donor and extend the life span of the acceptor signal, permitting the measurement of temporally delayed fluorescence.

The sensitivity of the assays for free β-hCG, PAPP-A, and PlGF was 0.16 IU/L, 0.004 IU/L, and 3.6 pg/mL, respectively. The intra (inter)-assay relative standard deviation for free β-hCG, PAPP-A, and PlGF was ≤4% (≤5%), ≤2% (≤4%), and ≤5% (≤7%), respectively.

### 4.3. Data Analysis

The daily multiple of median (dMoM) values of free β-hCG, PAPP-A, and PlGF were calculated. Gestational age-specific data normalization was carried out using the daily median curves generated from data kindly provided by Thermo Fisher Scientific, which were obtained using their KRYPTOR system from 222,475 patients for free β-hCG and PAPP-A, and 38,002 patients for PlGF. According to Thermo Fisher Scientific data, our daily free β-hCG and PAPP-A dMoM values were calculated for the gestational age range of 49–97 days (7–14 weeks) [87], while daily PlGF dMoM values were calculated for the gestational age range of 56–98 days (8–14 weeks) [88]. Only data within these ranges were used for the statistical calculations (free β-hCG and PAPP-A, *n*_RPL_ = 13, *n*_Control_ = 25; PlGF, *n*_RPL_ = 12, *n*_Control_ = 20). Since we did not have data for maternal weights, 69 kg was used in the equations as a general maternal weight [88].

To obtain classifiers based on free β-hCG, PAPP-A, and PlGF dMoM values, logistic regression models were trained using log2-transformed dMoM data, and the discriminative values of particular proteins and their combinations were investigated. Log2-transformed dMoM values were normalized for zero mean and unit standard deviation. A series of five-fold cross-validation procedures was performed with 50 random five-fold splits.

We also split samples into two subgroups, those with gestational age <9 and ≥9 weeks, respectively, resulting in the following per-protein sample sizes: for free β-hCG and PAPP-A, *n*_RPL_ = 8 (5), *n*_Control_ = 13 (12) for gestational ages <9 (≥9) weeks; for PlGF, *n*_RPL_ = 7 (5), *n*_Control_ = 8 (12) for gestational ages <9 (≥9) weeks, respectively. The same evaluation procedure was performed on the classifier trained on the whole data set and on two separate classifiers trained on the two subgroups (gestational age <9 and ≥9 weeks).

ROC curves were calculated for each protein separately as well as for all types of their combinations. The average ROC curve and the AUC values were determined from the 50 runs of cross-validation. Following clinical standards, we calculated the sensitivities (true-positive rates, TPRs), positive likelihood ratios, and negative likelihood ratios at 5% and 10% false-positive rates (FPRs) [184].

## 5. Conclusions

Our results show that free β-hCG and PAPP-A are good biomarkers for early RPL cases, and their discriminative power is even better for late RPL cases, while PlGF is a good marker for late RPL. The decreased maternal concentrations of these proteins indicate the deterioration of placental functions in RPL along with decreased placental angiogenesis in late RPL. In the future, larger prospective studies are needed for the investigation of whether these placental proteins also have predictive power for RPL before pregnancies fail.

## Figures and Tables

**Table 1:** Demographic and clinical data of the study groups.

Groups	RPL	Control
Number of cases ^a^	14	30
Maternal age (years) ^a^	37.2 ± 4.5 **	30.1 ± 7.0
Gestational age at surgery (weeks) ^a^	9.1 ± 1.9	8.6 ± 1.8
Gravidity ^b^	3 (2–4) *	2 (1–3)
Parity ^b^	1 (0–1)	0 (0–1)
Number of previous miscarriages ^b,c^	1 (1–1.8) ***	0 (0–0)

RPL: recurrent pregnancy loss; ^a^ values are presented as mean (standard deviation (SD)); ^b^ values are presented as medians (interquartile range (IQR)); ^c^ data were available for 29 cases in the control group; *** *p* < 0.001; ** *p* < 0.01; * *p* < 0.05 compared to gestational age-matched controls.

**Table 2:** Discriminative values of placental biomarker proteins.

	Concentration			dMoM		
	free β-hCG	PAPP-A	PlGF	free β-hCG	PAPP-A	PlGF
AUC	0.788	0.632	0.626	0.820	0.880	0.644
TPR % (5% FPR)	42.86	7.57	1.00	47.54	38.92	8.67
LR^+^ (5% FPR)	8.57	1.51	0.20	9.51	7.78	1.73
LR^−^ (5% FPR)	0.60	0.97	1.04	0.55	0.64	0.96
TPR % (10% FPR)	64.14	13.57	14.71	70.31	82.62	28.50
LR^+^ (10% FPR)	6.41	1.36	1.47	7.03	8.26	2.85
LR^−^ (10% FPR)	0.40	0.96	0.95	0.33	0.19	0.79

dMoM: daily multiple of median values; AUC: area under the curve (receiver operating characteristic (ROC) curve); TPR: true-positive rate/sensitivity; FPR: false-positive rate; LR^+^: positive likelihood ratio; LR^−^: negative likelihood ratio.

**Table 3:** Discriminative values of placental biomarker proteins in the gestational age range of 6–9 weeks.

	Concentration			dMoM		
	free β-hCG	PAPP-A	PlGF	free β-hCG	PAPP-A	PlGF
AUC	0.619	0.463	0.279	0.667	0.789	0.368
TPR % (5% FPR)	10.00	4.89	0.00	11.50	14.00	3.71
LR^+^ (5% FPR)	2.00	0.98	0.00	2.30	2.80	0.74
LR^−^ (5% FPR)	0.95	1.00	1.05	0.93	0.91	1.01
TPR % (10% FPR)	17.11	8.44	0.22	30.50	26.50	3.71
LR^+^ (10% FPR)	1.71	0.84	0.02	3.05	2.65	0.37
LR^−^ (10% FPR)	0.92	1.02	1.11	0.78	0.82	1.07

dMoM: daily multiple of median values; AUC: area under the curve (receiver operating characteristic (ROC) curve); TPR: true-positive rate/sensitivity; FPR: false-positive rate; LR^+^: positive likelihood ratio; LR^−^: negative likelihood ratio.

**Table 4:** Discriminative values of placental biomarker proteins in the gestational age range of 9–13 weeks.

	Concentration			dMoM		
	free β-hCG	PAPP-A	PlGF	free β-hCG	PAPP-A	PlGF
AUC	0.999	0.635	0.778	0.975	0.998	0.924
TPR % (5% FPR)	99.60	14.40	26.00	70.40	98.00	24.40
LR^+^ (5% FPR)	19.92	2.88	5.20	14.08	19.60	4.88
LR^−^ (5% FPR)	0.00	0.90	0.78	0.31	0.02	0.80
TPR % (10% FPR)	100.00	32.40	59.20	100.00	100.00	100.00
LR^+^ (10% FPR)	10.00	3.24	5.92	10.00	10.00	10.00
LR^−^ (10% FPR)	0.00	0.75	0.45	0.00	0.00	0.00

dMoM: daily multiple of median values; AUC: area under the curve (receiver operating characteristic (ROC) curve); TPR: true-positive rate/sensitivity; FPR: false-positive rate; LR^+^: positive likelihood ratio; LR^−^: negative likelihood ratio.

**Table 5:** Discriminative values of placental biomarker protein combinations for the 6–13 gestational week period.

		free β-hCG, PAPP-A	free β-hCG, PlGF	PAPP-A, PlGF	free β-hCG, PAPP-A, PlGF
concentration	AUC	0.806	0.784	0.695	0.793
	TPR % (5% FPR)	44.14	56.71	14.71	45.86
	LR^+^ (5% FPR)	8.83	11.34	2.94	9.17
	LR^−^ (5% FPR)	0.59	0.46	0.90	0.57
	TPR % (10% FPR)	61.29	65.00	21.43	55.86
	LR^+^ (10% FPR)	6.13	6.50	2.14	5.59
	LR^−^ (10% FPR)	0.43	0.39	0.87	0.49
dMoM	AUC	0.867	0.786	0.865	0.846
	TPR % (5% FPR)	54.00	56.50	46.33	43.67
	LR^+^ (5% FPR)	10.80	11.30	9.27	8.73
	LR^−^ (5% FPR)	0.48	0.46	0.56	0.59
	TPR % (10% FPR)	76.92	63.00	80.00	75.33
	LR^+^ (10% FPR)	7.69	6.30	8.00	7.53
	LR^−^ (10% FPR)	0.26	0.41	0.22	0.27

dMoM: daily multiple of median values; AUC: area under the curve (receiver operating characteristic (ROC) curve); TPR: true-positive rate/sensitivity; FPR: false-positive rate; LR^+^: positive likelihood ratio; LR^−^: negative likelihood ratio.

**Table 6:** Discriminative models are built using different proteins and protein combinations.

Independent Variables	$ln\frac{p_{RPL}}{1-p_{RPL}}$
$\log_{2}\left[free\beta -hCG\right]$	$-0.894-1.432\times \log_{2}\left[free\beta -hCG\right]$
$\log_{2}\left[PAPP-A\right]$	$-0.865-0.781\times \log_{2}\left[PAPP-A\right]$
$\log_{2}\left[PlGF\right]$	$-0.607-0.468\times \log_{2}\left[PlGF\right]$
$\log_{2}\left[free\beta -hCG\right],\log_{2}\left[PAPP-A\right]$	$-0.946-1.283\times \log_{2}\left[free\beta -hCG\right]-0.377\times \log_{2}\left[PAPP-A\right]$
$\log_{2}\left[free\beta -hCG\right],\log_{2}\left[PlGF\right]$	$-0.646-1.414\times \log_{2}\left[free\beta -hCG\right]-0.0826\times \log_{2}\left[PlGF\right]$
$\log_{2}\left[PAPP-A\right],\log_{2}\left[PlGF\right]$	$-0.672-0.795\times \log_{2}\left[PAPP-A\right]-0.111\times \log_{2}\left[PlGF\right]$
$\log_{2}\left[free\beta -hCG\right],\log_{2}\left[PAPP-A\right],\log_{2}\left[PlGF\right]$	$-0.695-1.287\times \log_{2}\left[free\beta -hCG\right]-0.542\times \log_{2}\left[PAPP-A\right]+0.134\times \log_{2}\left[PlGF\right]$
$\log_{2}\left({dMoM}_{free\beta -hCG}\right)$	$-0.740-1.552\times \log_{2}\left({dMoM}_{free\beta -hCG}\right)$
${{log}_{2}(dMoM}_{PAPP-A})$	$-0.842-1.782\times \log_{2}\left({dMoM}_{PAPP-A}\right)$
${{log}_{2}(dMoM}_{PlGF})$	$-0.379-0.720\times \log_{2}\left({dMoM}_{PlGF}\right)$
$\log_{2}\left({dMoM}_{free\beta -hCG}\right)$ $,\;{{log}_{2}(dMoM}_{PAPP-A})$	$-0.783-0.867\times {log}_{2}({dMoM}_{free\beta -hCG})-1.260\times {{log}_{2}(dMoM}_{PAPP-A})$
${{log}_{2}(dMoM}_{free\beta -hCG}),\log_{2}\left({dMoM}_{PlGF}\right)$	$-0.319-1.314\times {log}_{2}{(dMoM}_{free\beta -hCG})-0.292\times \log_{2}{(dMoM}_{PlGF})$
${{log}_{2}(dMoM}_{PAPP-A}),\;{log}_{2}({dMoM}_{PlGF})$	$-0.381-1.697\times \log_{2}\left({dMoM}_{PAPP-A}\right)-0.075\times {{log}_{2}(dMoM}_{PlGF})$
${{log}_{2}(dMoM}_{free\beta -hCG}$ $),\;{{log}_{2}(dMoM}_{PAPP-A}$ $),\;{{log}_{2}(dMoM}_{PlGF})$	$-0.283-0.763\times {{log}_{2}(dMoM}_{free\beta -hCG})-1.338\times \log_{2}\left({dMoM}_{PAPP-A}\right)+0.0215\times {{log}_{2}(dMoM}_{PlGF})$

## Data Availability

Data is available upon request.

## Supplementary Materials

The supporting information can be downloaded at: https://www.mdpi.com/article/10.3390/ijms25031865/s1. Supplementary Figure S1: Tissue-specific mRNA expression levels for the *CGB3*, *PGF*, and *PAPPA* genes (probes 205387_s_at, 209652_s_at, and 201981_at, respectively) from the GeneAtlas U133A data set. The data were downloaded from the BioGPS portal (https://biogps.org, accessed 14 December 2023). Error bars represent 95% confidence intervals. Supplementary Table S1: Daily median values of PAPP-A, β-hCG, and PlGF.
