# Supplementary figures and images for: Decreased Expression of Placental Proteins in Recurrent Pregnancy Loss: Functional Relevance and Diagnostic Value
Source: Int J Mol Sci. 2024 Feb 3;25(3):1865. doi: 10.3390/ijms25031865 (PMC10855863; doi:10.3390/ijms25031865)

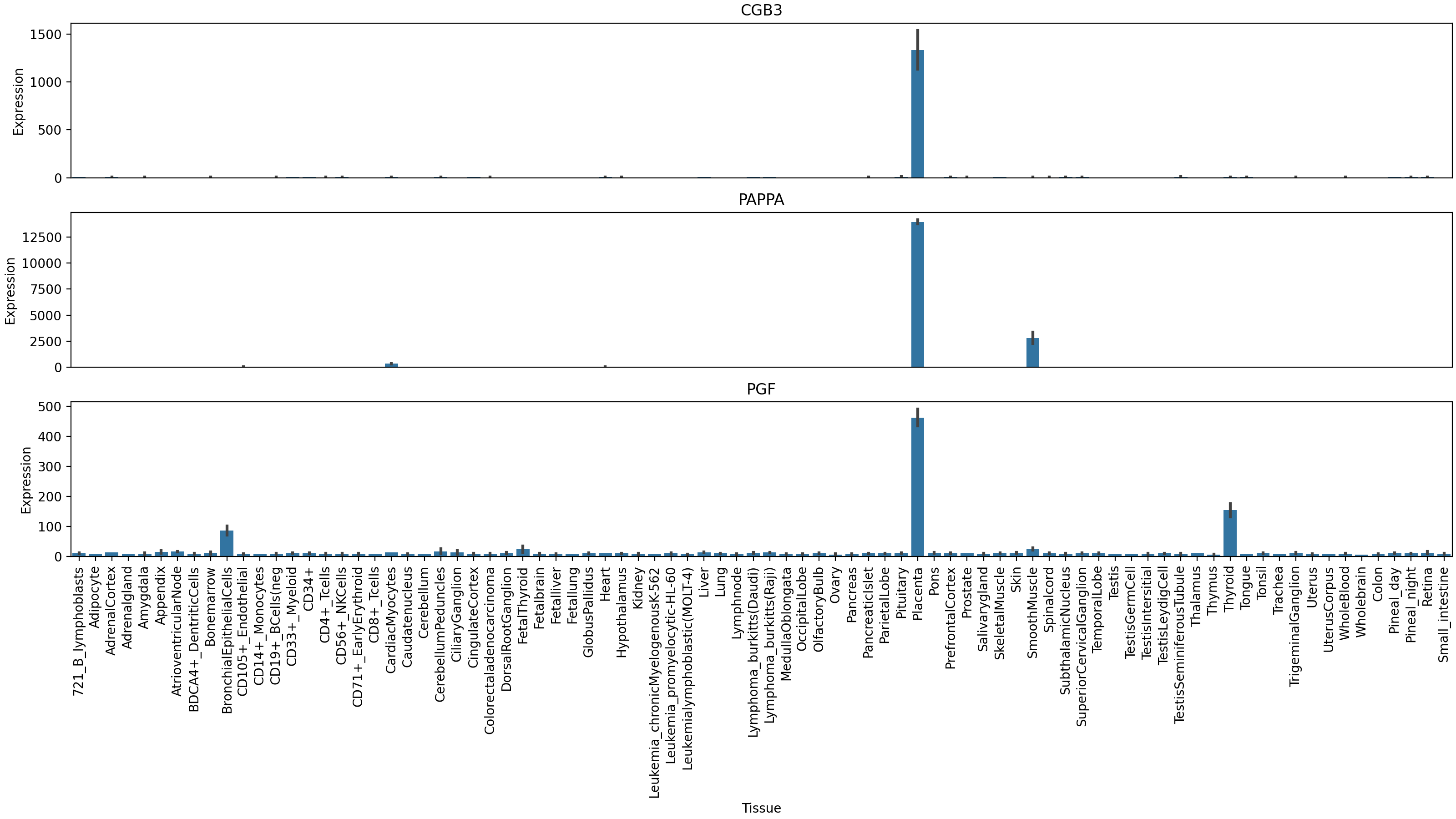

Supplement: Supplementary file 1 [file ijms-25-01865-s001.zip › Supplementary Figure S1.png]
